# A Compositive Strategy to Study the Pharmacokinetics of TCMs: Taking *Coptidis Rhizoma*, and *Coptidis Rhizoma*-*Glycyrrhizae Radix* et *Rhizoma* as Examples

**DOI:** 10.3390/molecules23082042

**Published:** 2018-08-15

**Authors:** Qiao Li, Yan Yang, Ting Zhou, Rui Wang, Na Li, Min Zheng, Yuan-Yuan Li, Ji-Quan Zhang, Fei Wu, Bai-Can Yang, Yue-Ming Ma, Bing-Liang Ma

**Affiliations:** 1 Department of Pharmacology, Shanghai University of Traditional Chinese Medicine, Shanghai 201203, China; cynnen@163.com (Q.L.); yangyan_gcdong@126.com (Y.Y.); zhouting718@163.com (T.Z.); zhengminpkyaodong@163.com (M.Z.); yuanyuan60107@163.com (Y.-Y.L.); 2 Engineering Research Center of Modern Preparation Technology of TCM of Ministry of Education, Shanghai University of Traditional Chinese Medicine, Shanghai 201203, China; 18265077559@163.com (R.W.); jiquan007@163.com (J.-Q.Z.); a1983d3891h@126.com (F.W.); 3 Department of Chinese Materia Medica, Shanghai University of Traditional Chinese Medicine, Shanghai 201203, China; licyoung@163.com (N.L.); bc_yang@163.com (B.-C.Y.)

**Keywords:** pharmacokinetic interaction, intestinal absorption, berberine, *Coptidis Rhizoma*, *Glycyrrhizae Radix et Rhizoma*

## Abstract

Pharmacokinetic studies are crucial for elucidating the effective constituents and formula compatibility of traditional Chinese medicines (TCMs). However, studies have usually been limited to single dosages and detection of systemic blood concentrations. To obtain comprehensive pharmacokinetic information, here we propose a multi-dosage and multi-sampling (blood from portal vein or systemic circulation, and liver) strategy to comparatively study the pharmacokinetics of multi-form TCMs, i.e., pure constituents, TCMs, or TCM formula extracts. Based on this strategy, we studied the pharmacokinetics of pure berberine, berberine in *Coptidis Rhizoma* (CRE), and berberine in *Coptidis Rhizoma*-*Glycyrrhizae Radix et Rhizoma* extracts (CR-GRE). After simple calculation and comparison of the obtained area under the curve (AUC) values, the results revealed the drastically different pharmacokinetic properties of pure berberine compared to CRE and CR-GRE. The results contribute to explaining the pharmacological loss of berberine activity after purification and the compatibility of the CR-GR drug pair. The results also innovatively showed that it was intestinal absorption that differentiated the pharmacokinetics of CRE and pure berberine, and CRE and CR-GRE. In conclusion, we propose a composite strategy to comparatively study the pharmacokinetics of TCMs, which could provide sufficient information to obtain a comprehensive view, before follow-up mechanism-of-action studies.

## 1. Introduction

Elucidating the effective constituents [1] and formula compatibility [2] is essential in traditional Chinese medicines (TCMs). With regard to effective constituents, it is surprising that the in vivo bioactivity of oral purified effective constituents is typically lower than the corresponding TCM extracts, as exemplified by artemisinin [3] and berberine [4]. In addition to the loss of other bioactive constituents, the decrease or loss of pharmacological effects of purified effective constituents is associated with the loss of synergic interactions among constituents after isolation and purification [5]. Moreover, interactions among constituents in formulated TCMs are also fundamental in terms of formula compatibility [2].

To elucidate interactions among constituents, the pharmacokinetics of TCMs have been extensively studied [6]. However, these studies have presented several limitations. Firstly, given that dose proportionality [7], an important pharmacokinetic property, plays crucial roles in guiding the clinical application of drugs, studies on the dose-exposure relationships that include multi-dosage pharmacokinetics of TCMs have been underrepresented. Secondly, studies on the pharmacokinetics and pharmacokinetic herb-herb and herb-drug interactions of TCMs have concentrated on drug metabolizing enzymes and transporters, like p-glycoprotein (P-gp) [6,8]. However, other mechanisms like solubility modification [9,10], tight junction opening [11], colloidal aggregates [12], or natural nanoparticle formation [4,13] may also be active in constituent interactions. Therefore, researchers need to have a robust understanding of the interactions before mechanistic studies are undertaken.

Here, we propose a composite strategy to comparatively study the pharmacokinetics of TCMs. In brief, we used a multi-dosage and multi-sampling (blood from portal vein or systemic circulation, and liver) strategy to study the pharmacokinetics of multi-form TCMs (pure constituents, TCMs, or TCM formula extracts). Based on simple calculation and comparison of the obtained AUC (area under the curve) values, we reasoned that substantial information conducive to building a comprehensive view could be provided before commencing follow-up mechanistic studies.

**Figure 1:**
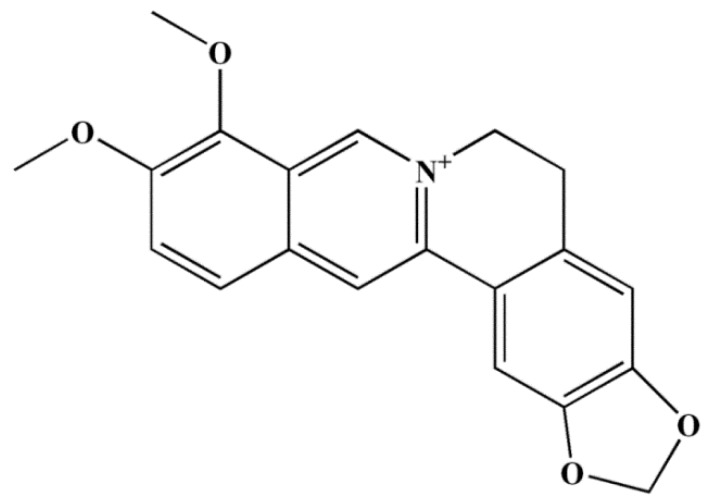
Structure of berberine.

*Coptidis Rhizoma*, a TCM produced from medicinal plants of the family Ranunculaceae like *Coptis chinensis* Franch, has been reported to have multiple pharmacological effects [14]. Isoquinoline alkaloids, and particularly berberine (Figure 1), have been identified as the major active constituents of *Coptidis Rhizoma* [14,15]. *Coptidis Rhizoma* is usually used in combination with other TCMs such as *Glycyrrhizae Radix et Rhizoma* [14], produced from the dried roots of plants of the family Leguminosae like *Glycyrrhiza uralensis* Fisch [16]. *Glycyrrhizae Radix et Rhizoma* is believed to counteract the toxicity associated with the use of combined toxic TCMs [16].

In this study, taking *Coptidis Rhizoma* and a *Coptidis Rhizoma-Glycyrrhizae Radix et Rhizoma* combination as examples, we aimed to evaluate the practicability of the proposed pharmacokinetic strategy. In addition, we ascertained whether this strategy would be useful in elucidating the mechanisms of pharmacological loss of berberine after purification and the compatibility of the *Coptidis Rhizoma*-*Glycyrrhizae Radix et Rhizoma* drug pair.

## 2. Results

### 2.1. Dose-Exposure Relationships of Orally Administered Pure Berberine, CRE, and CR-GRE

The whole PK parameters of berberine in pure berberine, CRE, and CR-GCE treated groups were shown in supplementary Table 1.

**Figure 2:**
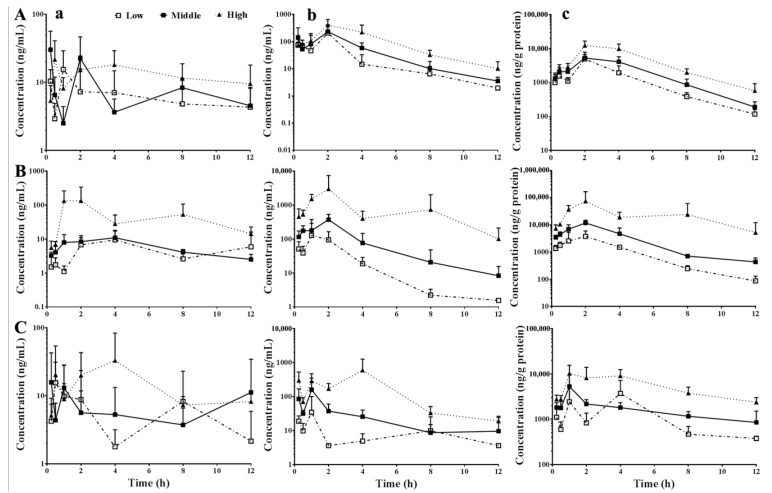
Dose-dependent concentration time curves of berberine in systemic circulation (**a**), portal vein (**b**), and liver (**c**) of mice that received oral pure berberine (**A**), *Coptidis Rhizoma* extract ((**B**), CRE), and *Coptidis Rhizoma*-*Glycyrrhizae Radix Et Rhizoma* extract ((**C**), CR-GRE), respectively (Mean ± SD, *n* = 6–8). Low, middle, and high indicates 58.7, 176.0, or 528.0 mg/kg pure berberine, or 0.33, 1.00, or 3.00 g/kg CRE, or 0.89, 2.67, or 8.00 g/kg CR-GRE, respectively. The dosages of pure berberine were equal to the contents of berberine in various dosages of CRE and CR-GRE, respectively.

The time-concentration curves of berberine in the systemic circulation, portal vein, and liver of mice administered various dosages of pure berberine, CRE, or CR-GRE are shown in Figure 2. The dose-related AUC_0–12h_ values and dose proportionality (γ^β-1^), calculated based on AUC_0–12h_ values, are listed in Table 1. The results showed that when the dosages were increased from low to high, the exposure levels of berberine in the systemic circulation, portal vein, and liver were sub-proportional (γ^β-1^ < 0.8) to the oral dosages of pure berberine, supra-proportional (γ^β-1^ > 1.25, except in the systemic circulation where 0.8 < γ^β-1^ < 1.25) to the oral CRE, and sub-proportional (γ^β-1^ < 0.8, except in the portal vein where γ^β-1^ > 1.25) to the oral CR-GRE. When the dosages were increased from middle to high, the exposure levels of berberine in the systemic circulation, portal vein, and liver were sub-proportional (γ^β-1^ < 0.8) to the oral dosages of pure berberine, but supra-proportional (γ^β-1^ > 1.25) to oral CRE. The exposure levels of berberine in the systemic circulation were sub-proportional (γ^β-1^ < 0.8) to the oral dosages of CR-GRE but were supra-proportional (γ^β-1^ > 1.25) in the portal vein and liver.

S. circulation indicates systemic circulation, P. vein indicates portal vein. Low, middle, and high indicate 58.7, 176, and 528 mg/kg pure berberine (ber), respectively, or three dosages of CRE (*Coptidis Rhizoma* extract) or CR-GRE (*Coptidis Rhizoma-Glycyrrhizae Radix et Rhizoma* extract), which contain corresponding dosages of berberine.

### 2.2. Hepatic Accumulation of Berberine

As shown in Table 2, the values of AUC_h_ were dozens of times higher than AUC_p_ and hundreds of times higher than AUC_c_, indicating the hepatic accumulation of berberine.

Low, middle, and high indicated 58.7, 176, and 528 mg/kg pure berberine (ber), respectively, or three dosages of CRE (*Coptidis Rhizoma* extract) or CR-GRE (*Coptidis Rhizoma-Glycyrrhizae Radix et Rhizoma* extract), which contained corresponding dosages of berberine. A_HP_ and A_HC_ indicated the ratios of hepatic exposure levels to portal vein exposure levels and hepatic exposure levels to systemic exposure levels, respectively.

### 2.3. Roles of Intestinal Absorption and Hepatic Disposition in the Dose-Exposure Relationships of Oral Pure Berberine, CRE, and CR-GRE

The F_L_ and F_I_ values of berberine in groups treated with various dosages of pure berberine, CRE, and CR-GRE are listed in Table 3. The F_L_ values decreased (the ratios of F_L-high_/F_L-low_ were smaller than 1) when the dosages of oral pure berberine, CRE, and CR-GRE increased from low to high, indicating that the hepatic disposal capacity was not reduced or saturated with an increase in dosage.

In addition, Table 3 shows that the F_I_ value decreased (the ratios of F_I-high_/F_I-low_ were smaller than 1) with the increase in dosage of oral pure berberine, indicating that the intestinal disposal capacity was reduced with the increase in dosage. However, the F_I_ values increased (the ratios of F_I-high_/F_I-low_ were larger than 1) in the CRE and CR-GRE treated groups, showing that the intestinal disposal capacity improved with increases in dosage.

Low, middle, and high indicated 58.7, 176, and 528 mg/kg pure berberine (ber), respectively, or three dosages of CRE (*Coptidis Rhizoma* extract) or CR-GRE (*Coptidis Rhizoma-Glycyrrhizae Radix et Rhizoma* extract), which contained corresponding dosages of berberine. F_L_ and F_I_ indicated the ratios of systemic exposure level to portal vein exposure level, and portal vein exposure level to oral dosages, respectively.

### 2.4. Roles of Intestinal Absorption and Hepatic Disposition in Differentiating the Pharmacokinetic Properties of Oral Pure Berberine, CRE, and CR-GRE

As shown in Table 3, the F_L_ values of pure berberine and CR-GRE (except for the low berberine dosage) treated groups were higher than CRE treated groups (the ratios of F_L-ber_/F_L-CRE_ or F_L-CR-GRE_/F_L-CRE_ were higher than 1), indicating that the hepatic disposal capacity in CRE treated groups was not reduced or saturated compared to groups treated with pure berberine or CR-GRE. Furthermore, Table 3 also shows that the F_I_ values of pure berberine and CR-GRE (except for the low berberine dosage) treated groups were substantially lower than CRE treated groups (the ratios of F_I-ber_/F_I-CRE_ or F_I-CR-GRE_/F_I-CRE_ were lower than 1), showing that the intestinal disposal capacity in CRE treated groups increased compared to groups treated with pure berberine or CR-GRE.

## 3. Discussion

Here, we report a composite strategy to study the pharmacokinetics of TCMs. Based on the proposed strategy, we analyzed the pharmacokinetic properties of pure berberine and berberine in CRE and CR-GRE; the results would then be further used to elucidate the mechanisms of loss of berberine activity after purification, as well as the compatibility of the CR-GR drug pair.

*Coptidis Rhizoma* has been reported to have an acute dose-toxicity relationship, which was attributed mainly to the contained berberine [15]. This previous study revealed that the exposure levels of berberine in mice receiving oral CRE were relatively high and supra-proportional to the oral CRE, providing a likely explanation for the observed dose-toxicity relationship. These pharmacokinetic phenomena have usually been explained as saturation of drug metabolism or elimination [17]. However, in this study, we found that it was the dose-dependently enhanced intestinal absorption of berberine in CRE that promoted the increase in the in vivo exposure levels. Co-existing factors have profound influences on the intestinal absorption of effective constituents in TCM extracts. As previously mentioned, potential mechanisms yet to be elucidated on a case-by-case basis include solubility modification [9,10], tight junction opening [11], colloidal aggregates [12], and natural nanoparticle formation [4,13]. Results from this study would stimulate further research into intestinal absorption and related mechanisms of action of effective constituents in TCM extracts. Indeed, naturally occurring proteinaceous nanoparticles in CRE were identified and shown to act as concentration-dependent carriers facilitating berberine absorption [4].

Compared with that of berberine in CRE, the in vivo pharmacological effects of pure berberine are limited; therefore, pharmaceutical [18] and chemical [19] techniques have been developed to promote its bioavailability and bioactivity. Correspondingly, it has been reported to have minimal toxicity and a poor dose-toxicity relationship [20]. This study revealed that the exposure levels of berberine were sub-proportional to its oral dosages. Based on the comparison of the dose-related F_I_ values, we suggest that poor intestinal absorption was crucial for its poor bioavailability and drug proportionality since the F_I_ values decreased with the increasing berberine dosage. Given that the solubility of berberine was limited [4], this implies that with a large dosage, the saturated solubility would restrict berberine intestinal absorption. This result was in accordance with reports that improving the solubility of berberine increased its exposure levels [21,22,23]. It is known that solubility is one of the fundamental factors controlling oral drug absorption [24], and various solubility-enhancing approaches have been developed to improve the solubility of oral drugs [25]. For better intestinal absorption, results from this study should promote the development of new strategies to increase the solubility of berberine, as well as other pure effective constituents.

Together with the pharmacokinetics of CRE, it is likely that intestinal absorption caused the pharmacokinetic differences between CRE and pure berberine, and these results explain the loss of berberine activity after isolation and purification.

In this study, hepatic disposition was crucial for the poor systemic exposure of oral pure berberine, since the F_L_ values were extremely low. Besides hepatic metabolism [26] and excretion [27,28], hepatic accumulation contributes greatly to the hepatic disposition of oral drugs. Our results showed that berberine accumulated significantly in the livers of mice that received oral pure berberine, CRE, or CR-GRE, which is in agreement with previous reports [15,26] and, furthermore, may explain why oral berberine shows certain in vivo pharmacological effects despite its extremely low systemic exposure levels. It has been reported that berberine may be actively taken up by hepatocytes via drug transporters such as organic cation transporters (OCTs) and organic anion transporting polypeptides (OATPs) [29]. Additionally, it was reported that berberine may be selectively distributed in subcellular organelles, especially mitochondria [30], which may also explain the hepatic accumulation of berberine. The results of this study showed that the F_L_ values of berberine in the livers of mice that received oral pure berberine, CRE, or CR-GRE decreased in a dose-dependent manner, indicating that the liver has a notable dose-dependent capacity to accumulate berberine. The results strongly suggest that hepatic accumulation played a decisive role in the hepatic disposition of berberine. Furthermore, the F_L_ values decreased with the increase in F_I_ values in groups treated with CRE and CR-GRE, suggesting the hepatic disposition of berberine was well correlated with that of intestinal absorption: the more absorbed, the more disposed.

Herb-mediated pharmacokinetic interactions have long been explained from the point of view of drug metabolizing enzymes and drug transporters [6,8]. In the case of *Glycyrrhizae Radix et Rhizoma* extract (GRE), it has been reported that some of its constituents, such as glycyrrhizin and glycyrrhetinic acid, may induce the drug metabolizing enzymes CYP3A and UGTs, while inhibiting the activity of efflux drug transporters including P-gp, multidrug resistance-associated proteins (MRPs), and breast cancer resistance protein (BCRP), and hence promoted herb-herb or herb-drug interactions [31]. In this study, the use of GRE significantly decreased the exposure levels of berberine in the systemic circulation, portal veins, and livers in mice administered oral CRE. Given the sharp dose-toxicity relationship associated with CRE, the results showed that *Glycyrrhizae Radix et Rhizoma* has both toxicity-reducing effects and harmonic activity, justifying its extensive use in TCM formulas [16]. Given that the F_L_ values in groups treated with CR-GRE were higher than with CRE, the effects of CR-GRE on the exposure levels of berberine in CRE were not mediated by the increase in berberine hepatic disposition. Since the F_I_ values were substantially lower in the CR-GRE compared to CRE-treated groups, the results suggest that the effects of CR-GRE were mainly due to decreased berberine intestinal absorption, as the mechanism could not be explained by its effects on intestinal drug transporters or drug metabolizing enzymes discussed above [31]. Inhibition of efflux transporter activity would conceivably lead to increased intestinal absorption of berberine, while enzyme induction would occur only after repeated administration. It has been reported that, besides low-molecular-weight constituents such as glycyrrhizin and glycyrrhetinic acid, GRE also contains constituents of high molecular weight such as polysaccharides; these may attract, and thus restrict, the release of effective compounds like aconitine and lead to a decrease in intestinal absorption [13]. Together with the pharmacokinetic results of CRE, we suggest that it was intestinal absorption that led to the pharmacokinetic differences between CRE and CR-GRE. These results should promote further research into intestinal absorption-based interactions between TCMs to elucidate formula compatibility.

Liu et al. [26] introduced an efficient pharmacokinetic strategy to study the first-pass elimination of berberine. In brief, berberine was administered via four different (i.e., intragastric, intraduodenal, intraportal, and intravenous) routes to anesthetized rats, and blood was then collected from the carotid artery at designated time points. After calculating and carefully comparing the four obtained AUC_0-t_ values of berberine in the carotid artery, they evaluated the contribution of gut and liver to the first-pass elimination of berberine. Our pharmacokinetic strategy has advantages over this strategy: (1) the tested drug was administered via a single route, i.e., oral administration; (2) several types of samples could be obtained, i.e., portal vein blood, systemic blood, and tissues such as liver; and (3) the experimental animals were not anesthetized until the samples were collected, as anesthesia may have a profound impact on drug pharmacokinetics. However, it is important to note that a large number of animals were used and, in addition, our pharmacokinetic strategy would not allow continuous blood sampling in an experimental animal. Consequently, the pharmacokinetic parameters could only be calculated based on average concentration values at designated time points; therefore, the upper or lower limits of the calculated pharmacokinetic parameters have not been provided in this study. However, we consider this to be an efficient strategy to use in pilot studies evaluating the pharmacokinetic properties of TCMs, given that it has the potential to simultaneously provide a substantial amount of information/amount of data.

## 4. Conclusions

In conclusion, we proposed a composite strategy to comparatively study the pharmacokinetics of TCMs to provide substantial information to obtain a comprehensive view before follow-up mechanistic studies are undertaken. Based on the proposed strategy, our data showed that intestinal absorption differentiated the pharmacokinetic properties of pure berberine from that of CRE and CR-GRE. We suggest that more studies are required on the intestinal absorption of TCMs as well as constituent interactions based on intestinal absorption.

## 5. Materials and Methods

### 5.1. Materials

*Coptidis Rhizoma* (*Coptis chinensis* Franch.) and *Glycyrrhizae Radix et Rhizoma* (*Glycyrrhiza uralensis* Fisch.) were purchased from the Shanghai Kang Qiao Herbal Pieces Co., Ltd. (Shanghai, China), a GMP-certificated manufacturer without any affiliation to the authors. The herbs were authenticated by Prof. Zhi-Li Zhao of the Department of Botany, Shanghai University of Traditional Chinese Medicine, according to *The Pharmacopoeia of People’s Republic of China* (2015 edition). Berberine hydrochloride and carbamazepine with purities >98% (used as standards) were obtained from the National Institute for the Control of Pharmaceutical and Biological Products (Beijing, China). Berberine hydrochloride with a purity >95% (used for oral administration in mice) was purchased from the Shanghai Yuan-Ye Biotechnology Co., Ltd. (Shanghai, China). Acetonitrile was purchased from Merck (Darmstadt, Germany). Ultra-pure water used in the current study was prepared using a Milli-Q purification system (Millipore Corporation, Billerica, MA, USA). All other materials were of analytical grade or higher.

### 5.2. Animals

Grade II male and female Kun-Ming (KM) mice (22–24 g) were purchased from the Shanghai Slac Laboratory Animal Co., Ltd. (Shanghai, China). The mice were housed in an air-conditioned room at 22–24 °C under a 12-h dark/light cycle and given food and water *ad libitum*. The mice were fasted overnight before the experiments. All animal experimental protocols were approved by the Institutional Animal Care and Use Committee of the Shanghai University of Traditional Chinese Medicine (Approval Number: 201708001), and all experiments were performed according to the guidelines established by this committee.

### 5.3. Preparation and Quality Control of Coptidis Rhizoma and Coptidis Rhizoma-Glycyrrhizae Radix et Rhizoma Extracts

The aqueous extract of *C. Rhizoma* was prepared as follows: briefly, the herbal portion of *C. Rhizoma* was extracted twice with boiled water (1.5 h for the first and 1 h for the second extraction). The extraction was filtered and dried under vacuum at 60 °C. The berberine content in the obtained powder (CRE) was determined to be 17.6%.

For the drug pair, the herbal portions of *C. Rhizoma* and *G. Radix et Rhizoma* (1:1) were extracted simultaneously and dried as described above. The ratio (1:1) of *C. Rhizoma* to *G. Radix et Rhizoma* was chosen according to their ratio in classic TCM formulas, such as *Huanglian* Decoction (with a 1:1 ratio), recorded in *Treatise on Cold Damage* (*Shang Han Lun*). The content of berberine in the obtained powder (CR-GRE) was determined to be 6.6%. The dried extracts were kept in an archive for further examination.

### 5.4. Liquid Chromatography Tandem Mass Spectrometry (LC-MS/MS)

Briefly, a Shimadzu Prominence UFLCXR series HPLC (Shimadzu, Kyoto, Japan) and a Thermo Scientific TSQ Quantum Ultra mass spectrometer (Thermo Scientific, Waltham, MA, USA) equipped with an electrospray ionization (ESI) source, were used. Carbamazepine was used as the internal standard. The samples were precipitated with three volumes of acetonitrile. After centrifugation (25,000× *g*, 10 min, 4 °C), the supernatant was mixed with an equal volume of water, and 10-μL samples were injected into the LC-MS/MS system. The samples were eluted through a Hypersil Gold (C18) analytical column (5 µm, 100 × 2.1 mm) with a gradient of the aqueous phase (0.08% *v*/*v* formic acid and 2 mM ammonium acetate) and the acetonitrile phase (0 min, 85:15; 7 min, 32:68; 7.01 min, 85:15; 10 min, 85:15) at a flow rate of 0.3 mL/min. The ESI source was set to positive ion mode. Data acquisition was performed in the multiple reaction monitoring mode of the selective mass transition for each compound. The transitions from the precursor ions to the protonated fragment product ions were monitored as follows: *m*/*z* 336.2 to *m*/*z* 322.3 for berberine, and *m*/*z* 237.00 to *m*/*z* 194.31 for carbamazepine. The linear dynamic range for berberine in the tested biological samples was 1.95 to 1000 ng/mL. The quality control samples were prepared at three different concentrations. The extracted ion chromatograms (EIC) of the internal standard and berberine were stacked and shown in supplementary Figures S1 and S2. The accuracy, precision, recovery, and stability tests all met the requirements of quantitative determination in biological samples and the method has previously been reported [21,32].

### 5.5. Pharmacokinetics of Berberine in Mice

Mice were randomly divided into groups (*n* = 6 or 8 mice per group). The mice were orally administered with various dosages of pure berberine (58.7, 176.0, or 528.0 mg/kg), CRE (0.33, 1.00, or 3.00 g/kg), and CR-GRE (0.89, 2.67, or 8.00 g/kg). The dosages of berberine were set based on but a little bit beyond its clinical applications (300–500 mg 3 times daily for an adult) [33] and preclinical studies using mice (100–300 mg/kg) [18] and were equal to the amounts of berberine in various dosages of CRE and CR-GRE. At the designated time points (0.25, 0.5, 1, 2, 4, 8, and 12 h), the mice were anesthetized with diethyl ether, and venous blood was sampled in succession from portal vein and posterior orbital venous plexus and collected into heparinized tubes. The plasma samples were obtained after centrifugation (6000× *g*, 10 min, 4 °C). After blood sampling, the livers were sampled and homogenized with pure water. The samples were stored at −80 °C for analysis using the validated LC-MS/MS method.

### 5.6. Pharmacokinetic Data Processing

The AUC values were calculated according to the trapezoidal rule and based on the mean values of drug concentrations at various time points. According to Equation (1) [26] [where F, F_a_, F_g_, and F_h_ indicate total absolute oral bioavailability, intestinal absorption, gastrointestinal and hepatic bioavailability, respectively], the amount of drug entering the systemic circulation (A) can be calculated as Equation (2), where A_ig_ indicates the dosage of the oral drug:

F = F_a_ × F_g_ × F_h_ (1)

A = A_ig_ × F_a_ × F_g_ × F_h_ (2)

Equation (2) can be further simplified to Equation (3):

A = A_ig_ × F_I_ × F_L_ (3)

F_I_ indicates intestinal disposal capacity, and can be calculated according to Equation (4), showing as the ratio of the drug that can enter the portal vein after intestinal disposition. AUC_p_ indicates the portal vein exposure levels of the oral drug. Larger F_I_ values indicate higher intestinal disposal capacity:

F_I_ = AUC_p_/A_ig_ (4)

F_L_ indicates hepatic disposal capacity, and can be calculated according to Equation (5), showing as the ratio of the drug that could enter from the portal vein to the systemic circulation. AUC_c_ indicates the systemic exposure levels of the oral drug. Smaller F_H_ values indicate higher hepatic disposal capacity:

F_L_= AUC_c_/AUC_p_ (5)

For comparative studies, Equations (6)–(8) can be used:

AUC_c1_/AUC_c2_ = A_ig1_/A_ig2_ × F_I1_/F_I2_ × F_L1_/F_L2_ (6)

F_I1_/F_I2_ = AUC_p1_/A_ig1_ × A_ig2_/AUC_p2_ (7)

F_L1_/F_L2_ = AUC_c1_/AUC_p1_ × AUC_p2_/AUC_c2_ (8)

Drug hepatic accumulation is displayed as A_HP_ and A_HC_, calculated according to Equations (9) and (10), respectively, where AUC_h_ indicates the hepatic exposure level of oral drugs. Larger A_LP_ and A_LC_ values indicate higher hepatic accumulation of oral drugs:

A_HP_ = AUC_h_/AUC_p_ (9)

A_HC_ = AUC_h_/AUC_c_ (10)

### 5.7. Statistical Analysis

Power model-based dose proportionality analysis was performed according to reference [7]. Deviation from dose proportionality was considered as relevant when the confidence interval for γ^β-1^ violates the interval of 0.8–1.25 [7].

## Figures and Tables

**Table 1:** AUC_0__–12h_ values and dose proportionality of berberine in mice that received oral berberine, CRE, and CR-GRE (*n* = 6–8).

TCMs	Locations	AUC_0–12h_ (ng·h/mL)			Proportionality (γ^β-1^)	
		Low	Middle	High	Low–High	Middle–High
Ber	S. circulation	75.4	99.2	158.7	0.23	0.28
	P. vein	462.1	685.9	1549.7	0.37	0.57
	Liver	16,673.0	26,711.9	60,648.4	0.40	0.57
CRE	S. circulation	62.6	75.3	629.8	1.10	7.76
	P. vein	333.6	1117.5	10,295.2	3.37	9.43
	Liver	14,359.6	43,657.3	311,370.2	2.37	5.65
CR-GRE	S. circulation	70.6	77.3	191.2	0.30	0.68
	P. vein	101.4	340.0	2537.0	2.78	6.19
	Liver	17,328.1	20,135.8	68,701.5	0.44	1.29

**Table 2:** Values of A_LP_ and A_LC_ in mice received various dosages of oral berberine, CRE, or CR-GRE (*n* = 6–8).

TCMs	Dosages	A_HP_	A_HC_
Ber	Low	36.1	221.1
	Middle	38.9	269.3
	High	39.1	382.2
CRE	Low	43.0	229.4
	Middle	39.1	579.8
	High	30.2	494.4
CR-GRE	Low	170.8	245.6
	Middle	59.2	260.6
	High	27.1	359.3

**Table 3:** Values of F_L_ and F_I_ in mice received various dosages of oral berberine, CRE, or CR-GRE (*n* = 6–8).

TCMs	Dosages	F_L_	F_L-high_/F_L-low_	F_L-ber_/F_L-CRE_ or F_L-CR-GRE_/F_L-CRE_	F_I_	F_I-high_/F_I-low_	F_I-ber_/F_I-CRE_ or F_I-CR-GRE_/F_I-CRE_
Ber	Low	0.16	/	0.87	462.1	/	1.39
	Middle	0.14	/	2.15	228.6	/	0.61
	High	0.10	0.63	1.67	172.2	0.37	0.15
CRE	Low	0.19	/	/	333.6	/	/
	Middle	0.07	/	/	372.5	/	/
	High	0.06	0.33	/	1143.9	3.43	/
CR-GRE	Low	0.70	/	3.71	101.4	/	0.30
	Middle	0.23	/	3.37	113.3	/	0.30
	High	0.08	0.11	1.23	281.9	2.78	0.25

## Supplementary Materials

Supplementary materials are available on line.
