# Supplementary material for: A Compositive Strategy to Study the Pharmacokinetics of TCMs: Taking Coptidis Rhizoma, and Coptidis Rhizoma-Glycyrrhizae Radix et Rhizoma as Examples
Source: Molecules. 2018 Aug 15;23(8):2042. doi: 10.3390/molecules23082042 (PMC6222803; doi:10.3390/molecules23082042)
Supplement: Supplementary file 1 [file molecules-23-02042-s001.pdf]

Supplementary table 1 The PK parameters of berberine in pure berberine, CRE, and CR-GCE treated groups (n=6-8)

TCMs	Locations	Dosages	PK parameters					
			C_{max} (ng/mL)	T_{max} (ng/mL)	$T_{1/2}$ (h)	MRT (h)	CL (mL/h)	AUC_{0-12} (ng·h/mL)
Ber	S.circulation	L	15.5	1.0	12.0	5.0	397.1	75.4
		M	30.3	0.3	7.1	5.0	1219.7	99.2
		H	21.7	0.5	8.6	5.5	1939.4	158.7
	P. vein	L	206.2	2.0	2.8	2.4	124.8	461.9
		M	231.8	2.0	2.0	2.7	253.3	685.8
		H	404.1	2.0	1.8	3.4	335.7	1549.7
	Liver	L	4951.2	2.0	2.0	3.3	3.5	16673.0
		M	5301.8	2.0	1.8	3.7	6.5	26711.9
		H	12333.8	2.0	1.9	3.9	8.5	60648.4
CRE	S.circulation	L	9.4	4.0	3.8	5.8	832.8	62.6
		M	11.0	4.0	5.1	4.7	1859.5	75.3
		H	133.8	2.0	8.2	4.6	611.6	629.7
	P. vein	L	128.1	1.0	1.7	2.2	174.8	333.6
		M	372.5	2.0	2.5	2.8	153.7	1117.5
		H	2989.2	2.0	4.0	4.0	47.1	10295.2
	Liver	L	3781.8	2.0	1.9	2.9	4.0	14359.6
		M	11891.3	2.0	2.3	3.0	3.9	43657.3

		H	75192.4	2.0	4.3	4.5	1.5	311370.2
CR-GRE	S.circulation	L	15.6	0.5	7.5	5.3	591.9	70.6
		M	15.8	0.3	9.8	3.2	1744.4	47.3
		H	33.2	4.0	3.5	4.7	2499.3	191.2
	P. vein	L	34.4	1.0	18.2	5.0	257.3	101.4
		M	159.5	1.0	4.7	3.1	450.6	340.0
		H	597.2	4.0	2.1	3.7	205.1	2537.0
	Liver	L	3710.0	4.0	2.4	4.2	3.2	17328.1
		M	5291.7	1.0	7.3	4.5	6.1	20135.8
		H	10254.8	1.0	4.1	4.6	6.4	68701.5

C_{max}, T_{max}, $T_{1/2}$, MRT, CL, $AUC_{0\text{-}12}$ indicate maximum concentration, time to C_{max}, elimination half-life, mean retention time, clearance, and the area under the concentration–time curve to the last measurable point (12 h), respectively.

S.circulation indicates systemic circulation, P. vein indicates portal vein. Low, middle, and high indicate 58.7, 176, and 528 mg/kg pure berberine (ber), respectively, or three dosages of CRE (*Coptidis Rhizoma* extract) or CR-GRE (*Coptidis Rhizoma-Glycyrrhizae Radix et Rhizoma* extract), which contain corresponding dosages of berberine.

It is important to note that the pharmacokinetic parameters were calculated based on average concentration values at designated time points; therefore, the upper or lower limits of the calculated pharmacokinetic parameters have not been provided in this study.

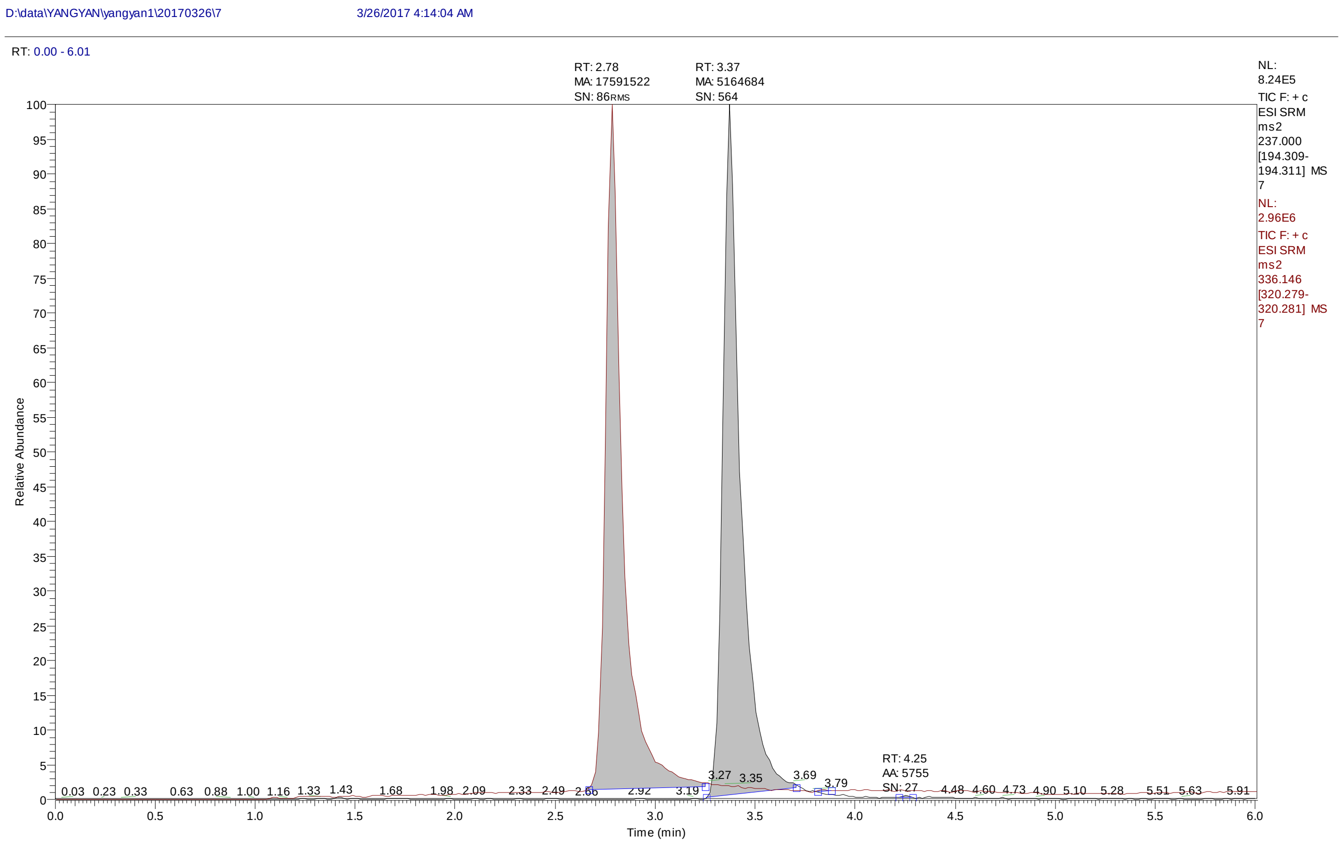

Supplementary Figure 1. Typical extracted ion chromatograms (EICs) of the mixed reference standards (left, berberine; right, the internal standard carbamazepine) or the carbamazepine spiked biological samples of mice treated with pure berberine.

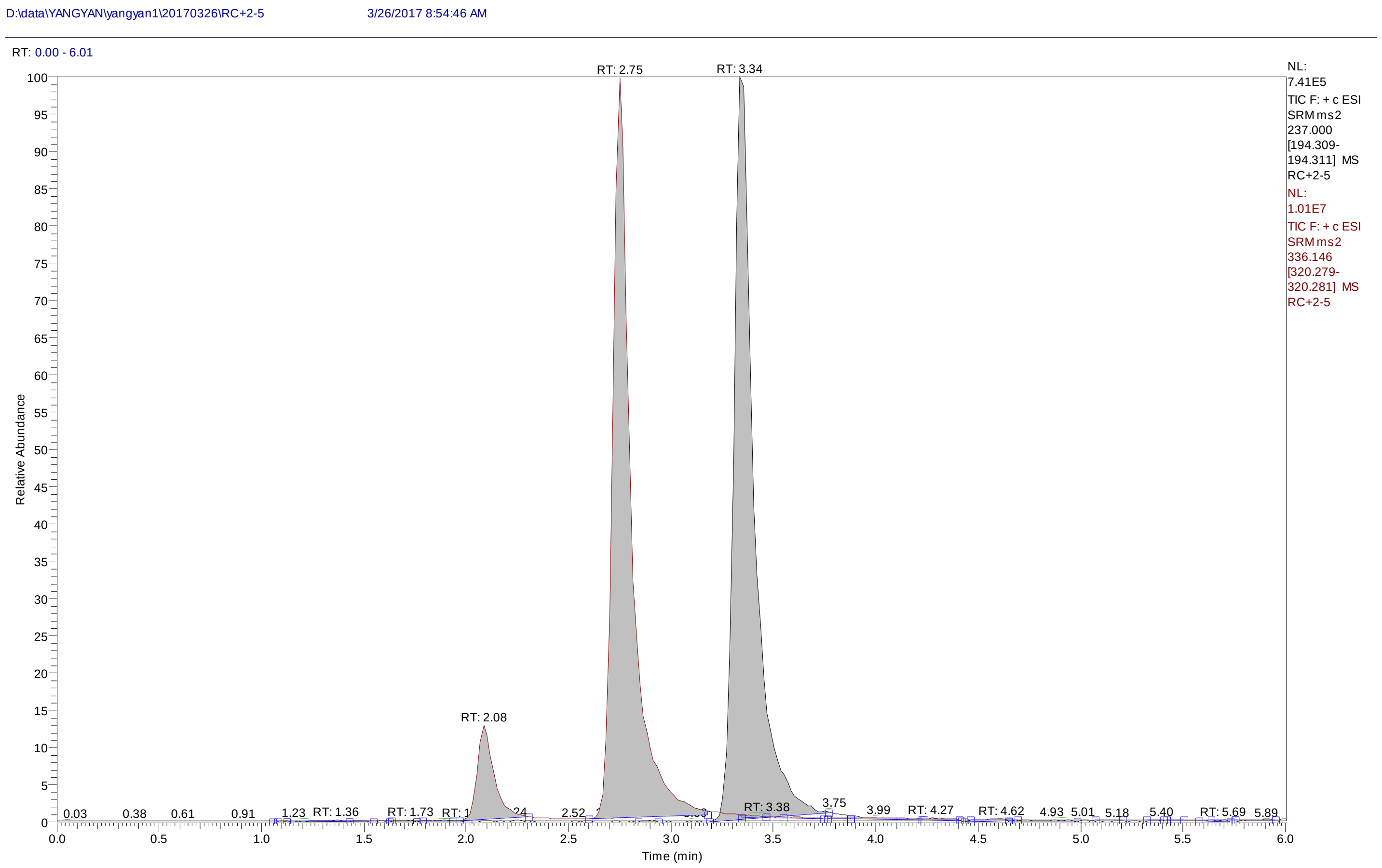

Supplementary Figure 2. Typical extracted ion chromatograms (EICs) of the carbamazepine spiked biological samples of mice treated with CRE or CR-GCE (left, epiberberine; middle, berberine; right, the internal standard carbamazepine).
